# A Case of Chronic Aortic Dissection With Hemopericardium and Tamponade

**DOI:** 10.7759/cureus.23924

**Published:** 2022-04-07

**Authors:** Athanasios Pavlou, Laura Cardenas Ramos, Martin Vanek, David J Regelmann

**Affiliations:** 1 Internal Medicine, St. Vincent's Medical Center, Bridgeport, USA; 2 Internal Medicine, Frank H. Netter MD School of Medicine, North Haven, USA

**Keywords:** echocardiography, orthopnea, hemopericardium, cardiac tamponade, aortic dissection

## Abstract

Type A aortic dissection involves the separation of the wall of the ascending aorta into a true lumen and a false lumen. The finding of an aortic dissection in a patient experiencing mild to moderate symptoms for several weeks may be surprising for clinicians, given the severity of the underlying process. Here, we present an 88-year-old patient who was admitted to our hospital due to orthopnea and leg swelling for the past two to three weeks and was found to have a chronic dissection of the ascending aorta, complicated by hemopericardium and tamponade. The existing literature very rarely reports chronic type A aortic dissection with tamponade on presentation.

## Introduction

Type A aortic dissection is an uncommon, life-threatening cardiovascular condition that is classically considered to be a surgical emergency. On rare occasions, type A aortic dissection is present in an asymptomatic or mildly symptomatic patient, deviating from the more common, rapidly progressive presentation. Regardless of the chronicity of the dissection, catastrophic complications can still occur at any point, leading to high morbidity and mortality rates.

## Case presentation

An 88-year-old female with a past medical history of cerebrovascular accident, chronic obstructive pulmonary disease, hypertension, hyperlipidemia, and prediabetes presented to our hospital with complaints of shortness of breath and lower extremity swelling. The patient reported that she was at her baseline until two to three weeks ago, when she noted gradual onset of shortness of breath, exacerbated by exertion, and orthopnea, which did not improve with the use of inhaled bronchodilators. At baseline she was able to ambulate around her home without limitations; however, she began experiencing progressive difficulty ambulating and required frequent breaks every 50 feet. During the same period, she also developed painless lower extremity swelling, left greater than right. She denied chest pain, palpitations, fever, chills, abdominal pain, nausea, vomiting, and leg pain. Due to these symptoms, the patient saw her cardiologist, who noted respiratory distress and referred her for hospital admission out of suspicion for an acute heart failure exacerbation. Transthoracic echocardiography (TTE) performed two months prior to her presentation demonstrated normal ejection fraction with mild to moderate aortic and tricuspid regurgitation as well as a small to moderate pericardial effusion. 

On arrival to the emergency department, she was afebrile, with a pulse of 74, respiratory rate of 20, blood pressure of 127/73, oxygen saturation of 94% on room air, and BMI 22.85 kg/m2. On the physical exam, the patient was ill-appearing but in no acute distress. Sparse wheezes and bibasilar rales were present as well as a new grade 2-3/6 systolic ejection murmur, best heard in the right upper sternal border, radiating to the bilateral carotids. Jugular venous distention and 2+ lower extremity pitting edema, greater on the left side were also present. The remainder of the physical exam including abdominal, neurological, and skin, were unremarkable. Complete blood count showed a hemoglobin level of 12.6g/dl and hematocrit of 37.7%. Blood chemistry was also unremarkable. Troponin levels measured at the time of admission were low (5 ng/L) and BNP was elevated at 182 pg/ml.

**Figure 1:**
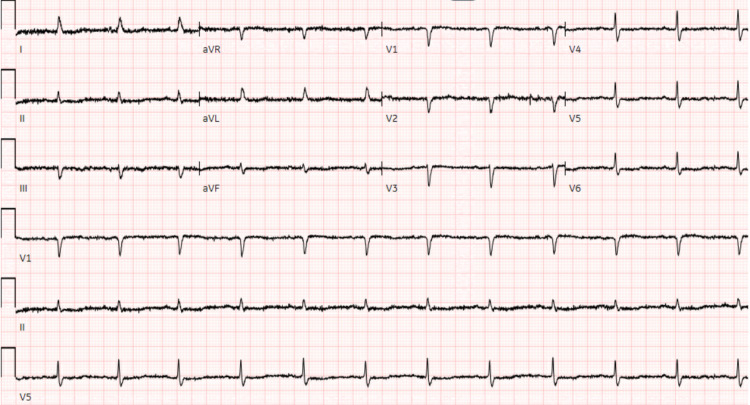
Electrocardiogram showing low QRS complexes.

**Figure 2:**
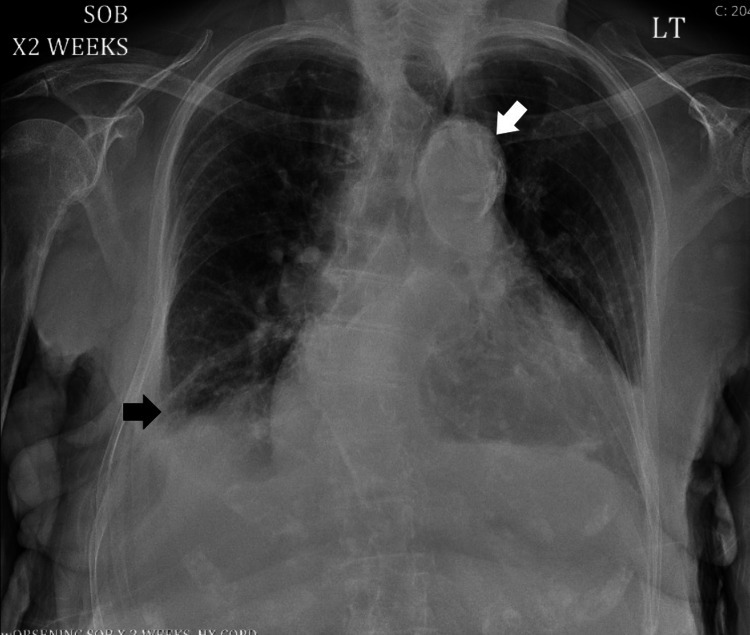
Chest radiograph showing a prominent and calcified aortic knob (white arrow). Additionally, a patchy right basilar opacity and a small right pleural effusion (black arrow) are seen.

Electrocardiogram demonstrated sinus rhythm, notable only for low voltage QRS complexes (Figure 1). Portable anteroposterior chest radiography showed a prominent, calcified aortic knob, cardiomegaly with mild pulmonary congestion, and patchy right basilar opacity with a small right pleural effusion (Figure 2). Concern for possible deep venous thrombosis given the patient’s asymmetric lower extremity edema prompted a left lower extremity duplex ultrasound, which showed no evidence of venous thrombi. Suspicion for pulmonary embolism was low (score of 0 on assessment with Wells’ criteria) [1], and the patient did not undergo computed tomography angiography (CTA). TTE was deferred to the following day, as the patient remained hemodynamically stable. The patient was admitted to the medical floors for the management of suspected acute exacerbation of congestive heart failure. She received treatment with 40 mg intravenous (IV) furosemide.

**Figure 3:**
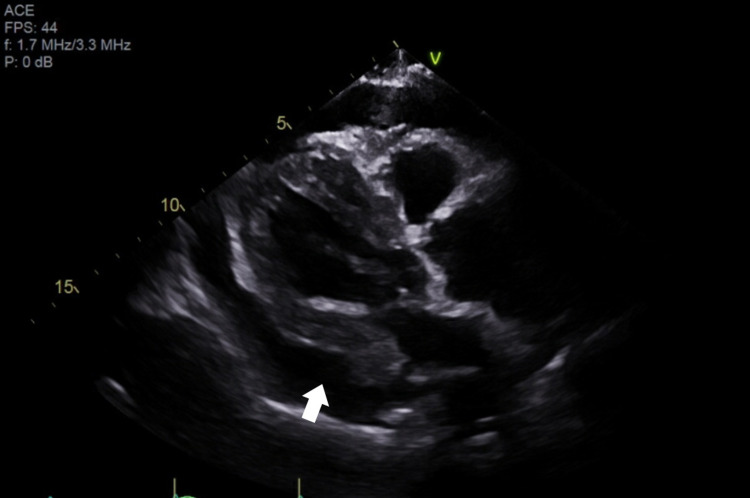
Long parasternal view of the heart on TTE showing large pericardial effusion. TTE: transthoracic echocardiography

**Figure 4:**
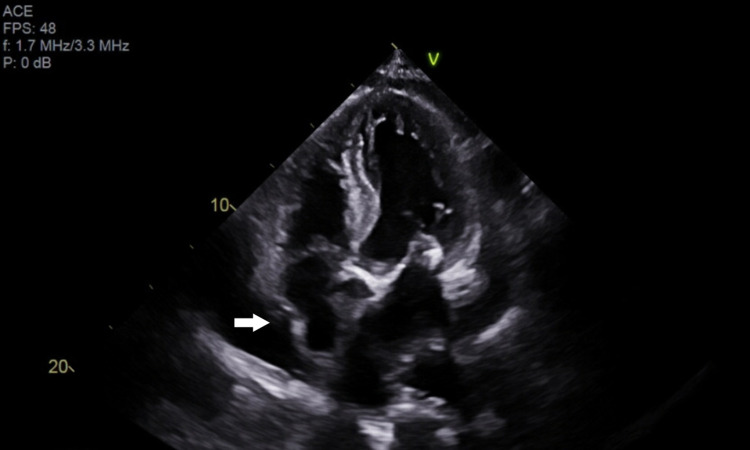
Apical four-chamber view of the heart on TTE showing inversion of the right atrium (a sign of tamponade). TTE: transthoracic echocardiography

**Figure 5:**
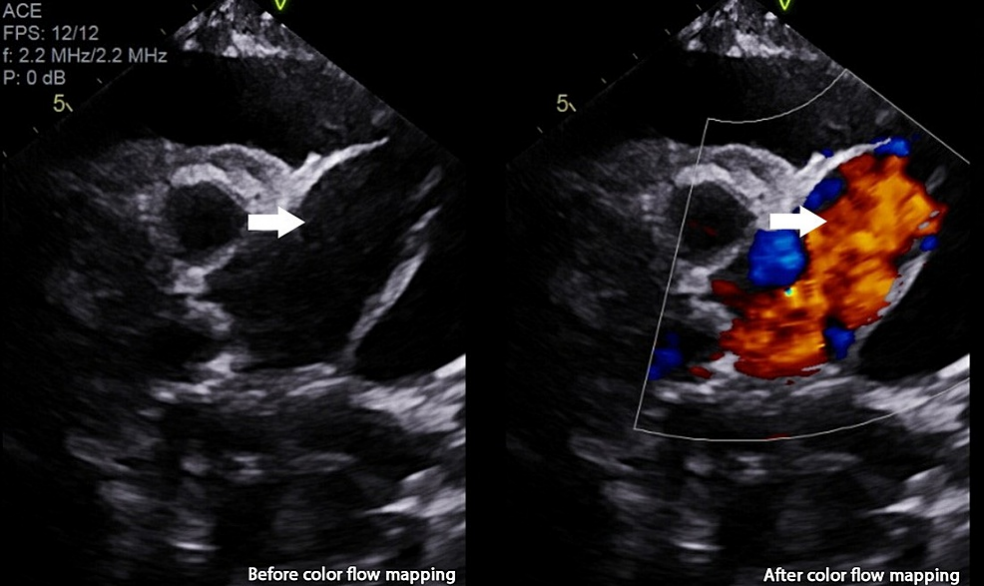
Aortic root on TTE seen severely dilated with dissection flap separating it into true and false lumen. On color Doppler imaging, blood is seen circulating within the true lumen. TTE: transthoracic echocardiography

**Figure 6:**
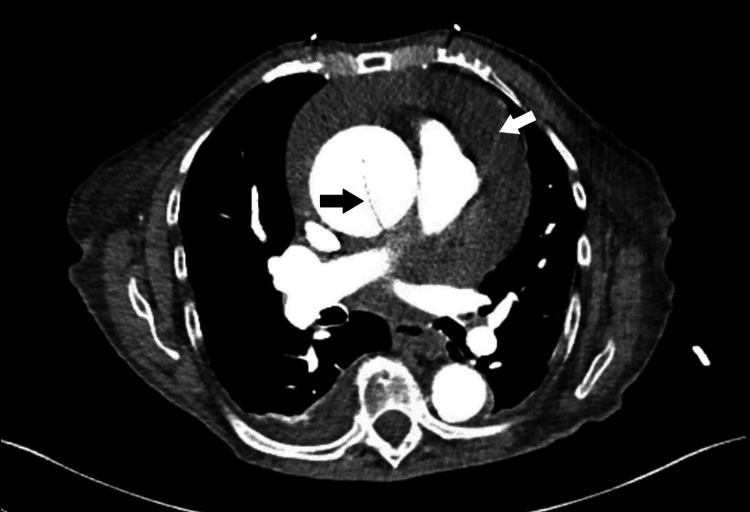
CTA of the chest demonstrating aneurysmal dilatation of the ascending thoracic aorta with a dissection flap extending to the proximal aortic arch (black arrow) as well as a large amount of hyperdense fluid within the pericardium suggestive of hemopericardium (white arrow). CTA: computed tomography angiography

**Figure 7:**
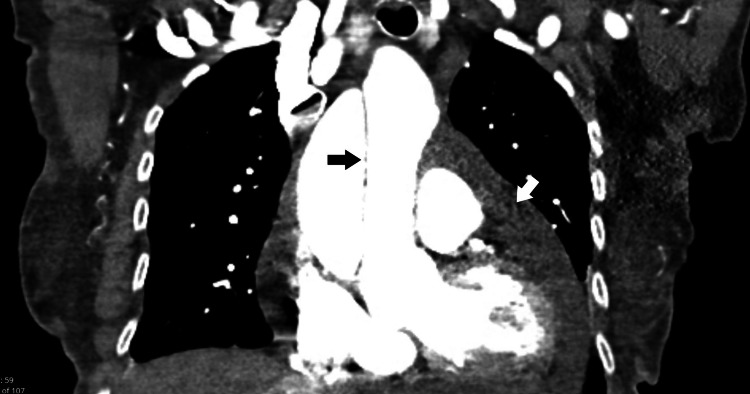
Coronal reconstruction of CTA showing extend of dissection (black arrow) to the aortic arch. Hemopericardium (white arrow) is also seen. CTA: computed tomography angiography

The following morning, the patient reported symptomatic improvement with resolution of shortness of breath at rest, but persistence during exertion. TTE showed a large circumferential pericardial effusion with areas of organization suggestive of thrombus and features suggestive of cardiac tamponade, such as right ventricular collapse, right atrial inversion, mild left atrial collapse, and plethoric inferior vena cava (Figure 3, Figure 4). TTE further demonstrated severe dilation of the ascending aorta at 5.7 cm and a linear echodensity within its proximal aspect, consistent with a dissection flap (Figure 5). As seen in Figure 6 and Figure 7, CTA of the chest, abdomen, and pelvis confirmed the presence of aneurysmal dilation of the ascending thoracic aorta with a dissection flap that extended to the proximal aortic arch. The descending thoracic and abdominal aorta were not involved. Further, a large collection of hyperdense fluid within the pericardium was consistent with hemopericardium.

Following the results of the imaging tests, the patient was emergently seen by the cardiothoracic surgery team, and surgical options were explored. However, she decided to avoid invasive interventions, opting instead for a conservative, palliative approach. The following week her condition deteriorated, and she expired eight days after her discharge.

## Discussion

Aortic dissection is a condition characterized by the separation of the wall of the aorta at the outer media or media-adventitia border, a process that starts with an intimal tear. Blood subsequently passes through the tear, moves in an anterograde or retrograde fashion, and dissects the lumen of the aorta into both true and false lumens [2]. Aortic dissections are classified according to location and acuity. The most commonly used classification is the Stanford system, which separates dissections that involve the ascending aorta (type A) from those that involve only the descending aorta (type B). The De Bakey system classifies dissections into type I, which involves the ascending aorta, aortic arch, and descending aorta, type II, which is confined to the ascending aorta, and type III, which is confined to the descending aorta distal to the left subclavian artery (rarely with retrograde extension) [3]. Stanford type A dissections are further classified into acute and chronic depending on the time of onset of initial symptoms to the time of presentation. If the interval between initial symptoms and presentation time is 14 days or less, then the dissection is considered acute. For an interval of more than 14 days, the dissection is considered chronic [4]. Another less frequently used classification system recommended by the European Society of Cardiology (ESC) subdivides the traditionally chronic (>14 days) into subacute (15-90 days) and chronic (>90 days) dissection [5].

A nationwide study estimated the incidence of aortic dissection at around 2.53 per 100,000 people per year, 66% of which corresponds to type A dissections. Classically, type A aortic dissection is considered a high mortality condition with a prehospital mortality rate of 24.8%. Among patients arriving alive at the hospital, the mortality rate is 28.9% within 24 hours of arrival [6]. Neurological deficits, altered mental status, myocardial and mesenteric ischemia, kidney failure, hypotension, cardiac tamponade, and limb ischemia are all possible complications of aortic dissection that contribute to the high in-hospital mortality [7].

A small subset of these patients presents with little to no symptoms and with a lack of severe complications. This presentation complicates the task of establishing the timing of the aortic dissection, with such dissections potentially remaining undetected for longer periods [8]. Available histopathologic data are scarce for chronic type A dissections, but several limited pathology reports allude to a possible mechanism for this unusual presentation. The development of a fully differentiated endothelial lining of the false lumen, changes in the collagen content and metalloproteinase expression, as well as the disruption of the elastic lamina, may alter the biomechanical behavior of the aorta, creating conditions that favor the stability of the wall and decelerate the expansion of the dissection [9,10].

Compared to the acute presentation, chronic type A dissections tend to be associated more frequently with a history of previous cardiac surgery, the presence of a bicuspid aortic valve, a larger ascending aortic diameter, higher rates of aortic root insufficiency, and less frequent extension beyond the aortic arch. These differences may indicate a distinct pathophysiologic mechanism or may be a consequence of low survival to the chronic phase among patients without the above characteristics [11]. Lyon et al. reported a decrease in cases of acute and an increase in cases of chronic aortic dissection, correlating to the onset of the coronavirus disease 2019 (COVID-19) pandemic, which was attributed to the delay in seeking medical advice [12]. This finding implies that at least some of the cases of chronic dissection may be the result of delayed diagnosis of a neglected acute dissection rather than a separate, less aggressive pathophysiologic process.

Management options for chronic type A aortic dissection include open surgery, which remains the gold standard, as well as endovascular repair or hybrid methods. Another acceptable approach is medical management. According to current guidelines, medical management remains an option for uncomplicated cases with an aortic diameter of less than 55mm [5]. Nevertheless, in a recent study by Kim et al. [13], the rate of adverse aortic events (aortic rupture and sudden death) remained significant at 12% at the five-year follow-up, which may suggest a greater benefit of the surgical approach. The main predictive factors for the occurrence of aortic events in this study were shown to be the baseline aortic diameter and patient age. In surgically managed chronic type A dissections, the mortality rate within 30 days from operation day is 6.1%, which is significantly lower than the corresponding rate for acute type A dissections [14]. Generally, chronic type A dissections have worse survival outcomes with conservative treatment compared to type B, which likely reflects the potential of more serious complications, such as involvement of the coronary vessels or pericardium, as well as the higher rate of expansion due to stronger dynamic forces closer to the heart [15].

Our patient experienced a chronic (or subacute according to the ESC system), Stanford type A, DeBakey type II dissection. Orthopnea and leg edema on presentation were consistent with the clinical appearance of acute congestive heart failure. The finding of dissection on both echocardiography and CTA was unexpected in the setting of mild to moderate symptoms and in the absence of chest pain. A clue to the underlying aortic pathology was the development of a new-onset systolic murmur in the right upper sternal border, radiating to the carotids, though such a murmur may have been attributed to aortic stenosis.

The presence of a large pericardial effusion with echocardiographic features of tamponade was a striking finding in our patient. The mechanisms for the development of such a pericardial effusion have been described in acute aortic dissections and involve either transudation through the wall of the false lumen or, less often, direct rupture of the dissection into the pericardial cavity. Hemodynamic compromise due to tamponade has more commonly been associated with the latter of these two mechanisms. [5]. Data on chronic dissections are limited and pericardial tamponade has very rarely been reported in the context of a chronic type A dissection. Fraser et al. in 1987 reported a case of chronic type A dissection that remained undetected for 16 years, causing only episodic chest pain before presenting with pericardial tamponade [16]. Yamaya et al. reported on a patient with cardiac tamponade secondary to ascending aortic dissection six weeks after a blunt trauma [17]. More recently, Makhoul et al. described a case of recurrent pericardial effusion with tamponade one month after an episode of severe chest pain, with further evaluation revealing an underlying dissection of the ascending aorta [18].

Our case is unique, as the patient was diagnosed with tamponade secondary to a spontaneous chronic type A aortic dissection on her very first presentation and without any history of chest pain. Remarkably, administration of low-dose diuretics led to significant but temporary symptomatic improvement. A possible explanation for this response lies in the chronicity of the dissection. If the dissection began roughly two to three weeks before presentation, blood may have progressively spread into the pericardial cavity through a low volume, contained rupture. Given the heterogeneous echogenicity and the hyperdense fluid on CTA, consistent with blood rather than serous fluid, transudation as a mechanism is less likely in our case. The resulting slow development of tamponade may have caused pulmonary congestion with increased central venous pressure, leading to orthopnea, lower extremity edema, and jugular venous distention, as seen on the patient’s physical exam, which initially responded to diuretic therapy.

According to the ESC guidelines [5], the aortic diameter of 5.7 cm and the presence of pericardial tamponade are clear indications for surgical intervention, had our patient consented to this approach. The care team avoided pericardiocentesis due to the high probability of fluid reaccumulation in the absence of a plan for definitive surgical correction.

## Conclusions

Chronic type A aortic dissection is an increasingly recognized entity. Whether this pathology represents a separate process or whether it is simply the outcome of surviving a latent acute event, clinicians will benefit their patients by maintaining awareness of its potential for fatal complications and by considering appropriate diagnostic and management options. A clinical presentation consistent with congestive heart failure and new-onset systolic aortic murmur indicates TTE evaluation. Conservative management may lead to a high level of morbidity and mortality and, thus, a surgical approach should be preferred in most cases, if it is consistent with the goals of care. A severely dilated aortic root and the presence of catastrophic complications, such as cardiac tamponade, should act as strong indications of the need for invasive procedures.

## References

[1] Wells PS, Anderson DR, Rodger M (2001). Excluding pulmonary embolism at the bedside without diagnostic imaging: management of patients with suspected pulmonary embolism presenting to the emergency department by using a simple clinical model and d-dimer. Ann Intern Med.

[2] Larson EW, Edwards WD (1984). Risk factors for aortic dissection: a necropsy study of 161 cases. Am J Cardiol.

[3] Tsai TT, Nienaber CA, Eagle KA (2005). Acute aortic syndromes. Circulation.

[4] Hynes CF, Greenberg MD, Sarin S, Trachiotis GD (2016). Chronic type A aortic dissection: two cases and a review of current management strategies. Aorta (Stamford).

[5] Erbel R, Aboyans V, Boileau C (2014). 2014 ESC guidelines on the diagnosis and treatment of aortic diseases: document covering acute and chronic aortic diseases of the thoracic and abdominal aorta of the adult. The Task Force for the Diagnosis and Treatment of Aortic Diseases of the European Society of Cardiology (ESC). Eur Heart J.

[6] Melvinsdottir IH, Lund SH, Agnarsson BA, Sigvaldason K, Gudbjartsson T, Geirsson A (2016). The incidence and mortality of acute thoracic aortic dissection: results from a whole nation study. Eur J Cardiothorac Surg.

[7] Mehta RH, Suzuki T, Hagan PG (2002). Predicting death in patients with acute type a aortic dissection. Circulation.

[8] Abugroun A, Subahi A, Gaznabi S, Daoud H (2019). Chronic type A aortic dissection: rare presentation of incidental pericardial effusion. Case Rep Cardiol.

[9] Carnevale D, Lembo G, Frati G (2011). Chronic type A aortic dissection: could surgical intervention be guided by molecular markers?. J Cell Mol Med.

[10] Emmott A, El-Hamamsy I, Leask RL (2017). Histopathological and biomechanical properties of the aortic wall in 2 patients with chronic type A aortic dissection. Cardiovasc Pathol.

[11] Rylski B, Milewski RK, Bavaria JE, Branchetti E, Vallabhajosyula P, Szeto WY, Desai ND (2015). Outcomes of surgery for chronic type A aortic dissection. Ann Thorac Surg.

[12] Lyon A, Gunga Z, Niclauss L, Rancati V, Tozzi P (2021). Case report: are we witnessing an increase of chronic ascending aortic dissection as a collateral effect to the COVID-19 pandemic?. Front Cardiovasc Med.

[13] Kim WK, Park SJ, Kim HJ, Kim HJ, Choo SJ, Kim JB (2019). The fate of unrepaired chronic type A aortic dissection. J Thorac Cardiovasc Surg.

[14] Wu J, Xie E, Qiu J (2020). Subacute/chronic type A aortic dissection: a retrospective cohort study. Eur J Cardiothorac Surg.

[15] Masuda Y, Yamada Z, Morooka N, Watanabe S, Inagaki Y (1991). Prognosis of patients with medically treated aortic dissections. Circulation.

[16] Fraser AG, Passani S, Hayward MW (1987). Chronic proximal aortic dissection presenting as tamponade after 16 years. Br Heart J.

[17] Yamaya K, Nitta Y, Yoshida S, Imamura Y, Takii T (2014). Traumatic type A dissection with acute pericarditis 6 weeks after blunt trauma; report of a case (Article in Japanese). Kyobu Geka.

[18] Makhoul M, Lorusso R, Bidar E, Zayad R, Natour E (2021). Misdiagnosed recurrent pericardial effusion in chronic type A aortic dissection. Isr Med Assoc J.

